# Bacterial infection and antibiotic resistance pattern in open fracture cases in Indonesia

**DOI:** 10.1016/j.amsu.2022.103510

**Published:** 2022-03-28

**Authors:** Ahmad Taufik, Adnanto Wiweko, Didit Yudhanto, E. Hagni Wardoyo, Philip Habib, Mohammad Rizki, Rohadi Muhammad Rosyidi

**Affiliations:** a Orthopaedic and Traumatology Subdivision, Department of Surgery, Medical Faculty of Mataram University, Mataram University Hospital, Mataram, Indonesia; b Doctorate Program, Faculty of Medicine, Hasanuddin University, Makassar, Indonesia; c Molecular Diagnostic Research Centre, Mataram University Hospital, Indonesia; d Department of Otolaryngology Head and Neck Surgery, Medical Faculty of Mataram University, Mataram, Indonesia; e Department of Clinical Microbiology, Medical Faculty of Mataram University, Mataram University Hospital, Mataram, Indonesia; f Department of Internal Medicine, Medical Faculty of Mataram University, Mataram University Hospital, Mataram, Indonesia; g Department of Clinical Pathology, Medical Faculty of Mataram University, Mataram University Hospital, Mataram, Indonesia; h Department of Neurosurgery Medical Faculty of Mataram University, West Nusa Tenggara General Hospital, Mataram, Indonesia

**Keywords:** Antibiotic, Infection, Osteomyelitis, Sensitivity test, Resistance pattern

## Abstract

**Background:**

The annual incidence of open fracture in Dr Soetomo Hospital, East Java were 400 cases with chronic infection complications exist in 14% (57 cases). A previous study in this hospital shows the resistance rate of Pseudomonas towards cefazolin and amikacin was 100% and 15%, respectively. The objective of this study was to identify bacterial infection type and antibiotic resistance pattern in infection caused by the open fracture.

**Methods:**

This was an analytic cross-sectional study. Samples were collected from three debridement surgery sites in Mataram Hospital, Mataram University Hospital, and Islamic Mataram Hospital from September 2019 until October 2020. Specimens from wound infection were cultured, and an antibiotic sensitivity test was performed.

**Results:**

Approximately 213 samples were analyzed in this study, comprising open fracture grade 3A (45%) and 3B (39%). The majority of fractures were lower extremity fractures (62%). Bacterial infection were found in 35% cases (80 isolates) in which 62,5% (50 isolates) were gram-positive bacteria and 37,5% (30 isolates) were gram-negative bacteria**.** Infection in open fracture was equivalent to grading. The predominant bacterial infection was caused by gram-positive bacteria, including *Staphylococcus aureus* and Staphylococcus negative coagulase. Gram-positive bacteria were sensitive towards Cepoferazone, Sulbactam and Ofloxacin, whereas gram-negative bacteria remains sensitive against Doxicyclin and Amicasin.

**Conclusion:**

Infection in open fracture was equivalent with the grade, and gram-positive were predominantly sensitive with cefoperazone sulbactam.

## Highlights

• This study was to identify bacterial infection type and antibiotic resistance pattern in infection caused by the open fracture.
• This was an analytic cross-sectional study.
• The predominant bacterial infection was caused by gram-positive bacteria, including Staphylococcus aureus and Staphylococcus negative coagulase.
• Infection in open fracture was equivalent with the grade, and gram-positive were predominantly sensitive with cefoperazone sulbactam.

## 1 Introduction

Open fractures due to trauma are still a significant problem in orthopaedics. Handling open fractures requires a longer treatment time and high costs, especially if a chronic infection [1]. At the hospital, dr. Soetomo Surabaya found cases of open fractures more than 400 cases each year, with the incidence of chronic disease is 57 cases [1,2].

The incidence of infection in open fractures is still high even though the treatment methods have been so advanced. Many studies show that grade 3 open fractures have the highest incidence of infection [3]. Patzakis reported an infection incidence of 0%–2% for type I, 2%–5% for type II, 5%–10% for type IIIA, 10%–50% for type IIIB and 25%–50% for type IIIC [4]. At Dr Soetomo Hospital between January 2009–December 2010, it was found that 50.4% were grade 3 open fractures [1].

Many studies from various countries have reported resistance to cefazolin and amikacin as standard antibiotic regimens for prophylactic therapy in open fractures. Johnson reported the incidence of methicillin-resistant *Staphylococcus aureus* in Texas during the 1980s [5]. Arcilla et al. reported Staphylococcus epidermidis resistant to Ampicillin, Penicillin, Cefazolin and Chloramphenicol in post-implantation osteomyelitis patients [25,26]. MH. Perlin and SA Lerner also wrote Amikacin resistance in E. Coli. In the last decade, there have been many reports of multi-drug resistance (methicillin, vancomycin, third-generation cephalosporins and fluoroquinolones) in *Staphylococcus aureus*, coagulase-negative Staphylococcus, *Pseudomonas aeruginosa*, and *Escherichia coli* [6,7].

In Herlambang's research at the Emergency Room (IRD), dr. Soetomo Hospital Surabaya found that the pattern of bacteria before debridement was carried out, gram-positive bacteria dominated the picture of germs. These bacteria were mainly *Staphylococcus aureus* 49.23% and *Pseudomonas aeruginosa* 20%, whereas, after debridement, the most common gram-negative bacteria were *Pseudomonas aeruginosa* (43.75%) and *Staphylococcus aureus* 18.75%. It was also found that resistance to cefazolin was 19.4% in *Staphylococcus aureus* and 100% in Pseudomonas. Resistance to amikacin was 10% in *Pseudomonas aeruginosa* and 5% in *Staphylococcus aureus* [8].

Before debridement, most bacteria were gram-positive, while gram-negative bacteria were dominant [21,22]. Other studies have also shown that gram-negative bacteria cause most infections in open fractures. In culture, it was found that the bacteria causing the condition did not reflect the bacteria that caused the contamination in open fractures when the patient came [28,29]. It was found that 92% of the causes of infection were nosocomial infections [7]. Based on this, the use of antibiotics cefazolin and amikacin in subsequent treatment in the room for up to 5 days needs to be re-examined. This is because the evaluation two days after debridement found resistance to standard antibiotics [8]. Researchers suspect that resistance to antibiotics is even higher after the patient is treated in the room. This study aims to determine the pattern of bacteria and their resistance to antibiotics in patients with open fractures undergoing orthopaedic surgery.

## 2 Method

This study is a cross-sectional analytic study in which data was collected only once. This study took samples from surgical debridement of infected patients with open fractures [23,27]. Then culture and sensitivity test (sensitivity) to antibiotics was carried out. This study selected all patients with musculoskeletal infections due to open fractures treated at the Mataram City Hospital, Mataram University Hospital and Siti Hajar Islamic Hospital Mataram from September 2019 to October 2020. Bacterial colonies were grown by planting infected specimens on blood agar and Mac Conkey culture media [30,31]. The pattern of antibiotic resistance was checked by administering an antibiotic plate on the bacterial culture medium. You will see a colony-free area on the culture if the antibiotic is effective against the bacteria.

## 3 Result and discussion

### 3.1 Characteristics of research data

In 1 year (September 2019–October 2020), 213 musculoskeletal culture specimens were obtained from patients at the University Hospital of Mataram, Mataram City Hospital and Siti Hajar Islamic Hospital Mataram. These cultures were obtained from surgical debridement in a patient with a musculoskeletal infection in an open fracture (Table 1).

**Table 1:** Characteristic of patients with musculoskeletal infection.

No	Patient Characteristics	Amount	Percentage (%)
**Gender**			
**1**	Male	137	64.3
	Female	76	35.7
	Total	213	100
**Age**			
**2**	<10 years	3	1
	10–20 years	44	21
	21–40 years	115	54
	41–50 years	43	20
	>50 years	8	4
	Total	213	100
**Causes of Infection**			
**3**	Work accident	20	9
	Traffic accident	147	69
	Accident at home	46	22
	Total	213	100
**Open Fracture Grade**			
**4**	Grade 1	8	4
	Grade 2	24	11
	Grade 3A	96	45
	Grade 3B	82	39
	Grade 3C	3	1
	Total	213	100
**Fracture Location**			
**5**	Upper extremity	74	35
	Lower Extremities	133	62
	Pelvis and Spine	6	3
	Total	213	100

Source: Data proceed.

Of the 213 cases of musculoskeletal infection that were swab and cultured, 137 (64.3%) were men, and 115 patients (54%) were aged between 21 and 40 years. Most cases were traffic accidents (59%). The most were open fracture grades 3A (45%) and 3B (39%) in all total cases. The most common fracture sites were in the lower extremities (62%).

### 3.2 Characteristics of the results of bacterial culture examination

In the span of 1 year (September 2019–October 2020), there were 213 cultures of pus specimens/wound bed swabs in cases of musculoskeletal infection. Of the 213 illustrations, 133 (65%) of them had no bacterial growth, and 80 isolates (35%) grew with the predominance of gram-positive bacteria, 50 isolates (62.5%) and gram-negative bacteria 30 isolates (37.5%). Seen in Table 2:

**Table 2:** Bacteria that cause musculoskeletal infections.

Organism	n	%
*Staphylococcus aureus*	26	32.5
Staphylococcus coagulase-negative	18	22.5
Staphylococcus epidermidis	6	7.5
*Escherichia coli*	14	17.5
Klebsiella aerogenes	2	2.5
*Enterobacter cloacae*	4	5
Pseudomonas aeroginosa	4	5
Pseudomonas species	3	3.75
Klebsiella sp	2	2.5
*Klebsiella pneumoniae*	1	1.25
	80	100

### 3.3 Overview of antibiotic sensitivity

#### 3.3.1 The sensitivity of the group of gram-positive cocci to antibiotics (n = 50)

**Fig. 1:**
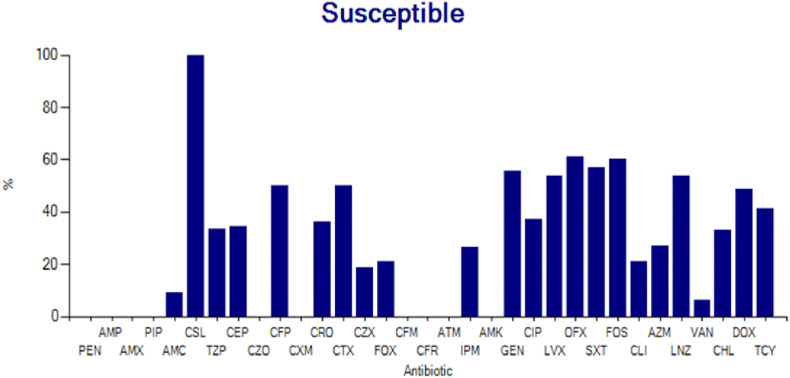
Bar diagram of the sensitivity of Gram-Positive Cocci to several antibiotics.

The bacteria included in this group in this study were *Staphylococcus aureus*, Staphylococcus coagulase-negative, Staphylococcus epidermidis. Here is the sensitivity to antibiotics (Fig. 1).

From the picture above, it can be seen that the sensitivity of gram-positive cocci to antibiotics with a sensitivity of 50% or more, from the highest to the lowest, were cefoperazone sulbactam (100%), ofloxacin (61%), fosfomycin (60%), trimethoprim-sulfamethoxazole (59%). %), gentamicin (58%), linezolid (57%), cefoperazone, cefotaxime and doxycycline with similar results 53% and tetracycline (50%).

#### 3.3.2 Sensitivity of gram-negative bacteria group to antibiotics (n = 30)

**Fig. 2:**
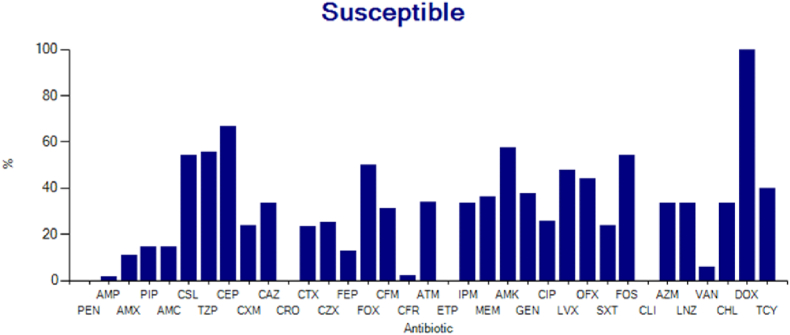
Bar diagram of the sensitivity of gram-negative rods to several antibiotics.

From Fig. 2 above, it can be seen that the sensitivity of gram-negative bacilli to antibiotics in musculoskeletal infections with a sensitivity of 50% or more, from the highest to the lowest are doxycycline. (100%), cephalothin (68%), amikacin (62%), piperacillin/tazobactam. (57%), cefoperazone-sulbactam (56%), cefoxitin (53%), and levofloxacin (50%).

## 4 Discussion

In this study, the highest incidence of infection was found in grade IIIA (45%) and IIIB (39%). The results follow the study results by Ref. [18]^,^ who also got the most infections in grade III (80%) with grade IIIA as much as 46.67%. Research in Brazil also got almost the same results; as many as 72% of infections occurred in grade III; in grade II, there were 24% infections, and grade I only 4% [19]. These results are consistent with the results of previous studies that the incidence of disease in open fractures will increase according to the increasing severity of the open fracture degree [9,10].

In this study, 50 isolates (62.5%) of gram-positive cocci and 30 isolates of gram-negative bacilli (37.5%) were found. The dominant gram-positive bacteria were *Staphylococcus aureus* (32.5%) and coagulase-negative Staphylococcus (22.5%). The gram negative bacteria are Pseudomonas aeroginosa, E Colli and Klebsiella sp [24]. These results are consistent with Jember, East Java, where 66.67% consisted of gram-positive bacteria, and 33.33% were gram-negative bacteria. The dominant bacteria is coagulase-negative staphylococcus [11,12].

These results are somewhat different from the research results by Ref. [11]^,^ where the results of the culture are mostly gram-negative bacteria. The dominant bacteria are Acinetobacter, Pseudomonas, Enterobacter, and *Escherichia coli*. Ninety per cent of the cultures were gram-negative bacteria, indicating that nosocomial infections occurred because the types of bacteria found were different from the patient's culture results when he first came to the hospital. The study results in India also got the same results where most of the cultures showed gram-negative bacteria [13,14].

Antibiotics that were found to be sensitive to these gram-positive bacteria were Cepoferazone sulbactam, ofloxacin and fosfomycin. For gram-negative bacteria, Sensitive antibiotics are doxycycline, cephalothin and amikacin. A study by Ref. [13] found all gram-positive bacteria showed low resistance (<60%) to antibiotics except ampicillin and penicillin (60–80%). Almost all gram-positive bacteria showed multiple drug resistance (52.7%). All Clostridium spp. were sensitive to tetracycline, doxycycline and kanamycin and had low resistance (<60%) to chloramphenicol, clindamycin and penicillin [14]. All gram-negative showed low resistance (<60%) to antibiotics except ampicillin and amoxicillin (60–80%). Fifty-one per cent of gram-negative bacteria were identified as multiple drug-resistant (MDR) [17] ^[180^.

Various studies have shown that gram-negative and gram-positive bacteria always cause infection in open fractures. Based on this, the antibiotics given must be able to eliminate these two types of bacteria. Aminoglycosides are effective antibiotics to treat both types of bacteria. Other studies have shown that Ciprofloxacin, norfloxacin and gentamicin are effective antibiotics to treat these gram-negative and gram-positive bacteria [19,20]. The administration of cephalosporins or quinolones should be combined with aminoglycosides in all cases of open fractures to increase their effectiveness [21,22].

## 5 Conclusion

In this study, it was found that infection in open fractures is directly proportional to the severity of the fracture grade that occurs. Most of the bacteria that cause infection are gram-positive bacteria, with the dominant bacteria being staphylococcus aureus and coagulase-negative staphylococci. Antibiotics sensitive to positive bacteria are cefoperazone sulbactam and ofloxacin, while doxycycline and amikacin are for gram-negative bacteria.

## Ethical approval

All procedure for research has been approved by the ethics committee of Mataram University Hospital.

## Sources of funding

No funding or sponsorship.

## Author contribution

ATF, ADW, DDY, EHW, PHB, MOR and RHA wrote the manuscript and participated in the study design. ATF, ADW, DDY, EHW, PHB, MOR and RHA drafted and revised the manuscript. ATF, and EHW performed treatment and surgery of Open Fracture with debridement surgery sites infection. ATF, ADW, DDY, EHW, PHB, MOR and RHA performed bioinformatics analyses and revised the manuscript. All authors read and approved the final manuscript.

## Registration of research studies

1. Name of the registry: http://www.researchregistry.com. Registration Date: January 14, 2022 12:35.

2. Unique Identifying number or registration ID: researchregistry7546.

3. Hyperlink to your specific registration (must be publicly accessible and will be checked): https://www.researchregistry.com/register-now#home/registrationdetails/61e16e1543fed1001e261f84/

## Guarantor

Rohadi Muhammad Rosyidi.

## Consent

This manuscript data from medical record patients diagnosed Open Fracture with debridement surgery sites Infection in Mataram Hospital, Mataram University Hospital, and Islamic Mataram Hospital from September 2019 until October 2020.

## Declaration of competing interest

The authors declare that they have no conflict of interests.
